# Alteration of Gastrointestinal Function and the Ameliorative Effects of *Hericium erinaceus* Polysaccharides in Tail Suspension Rats

**DOI:** 10.3390/nu17040724

**Published:** 2025-02-18

**Authors:** Peng Zang, Pu Chen, Junli Chen, Jingchao Sun, Haiyun Lan, Haisheng Dong, Wei Liu, Nan Xu, Weiran Wang, Lingwei Hou, Bowen Sun, Lujia Zhang, Jiaqiang Huang, Pengjie Wang, Fazheng Ren, Siyuan Liu

**Affiliations:** 1 College of Food Science & Nutritional Engineering, China Agricultural University, Beijing 100083, China; zp643027@163.com (P.Z.); renfazheng@263.net (F.R.); 2 National Key Laboratory of Space Medicine, China Astronaut Research and Training Center, Beijing 100094, China; chenpucpo@163.com (P.C.); jyjunli@163.com (J.C.); sjc964531@163.com (J.S.); lanhaiyun@163.com (H.L.); dhs303@126.com (H.D.); laoshuxizao@163.com (W.L.); 15504572568@163.com (N.X.); wrwang997@163.com (W.W.); houlingweihn@163.com (L.H.); sunbowenfood@163.com (B.S.); lujiazhang0118@163.com (L.Z.); 3 Department of Nutrition and Health, China Agricultural University, Beijing 100193, China; wpj1019@cau.edu.cn

**Keywords:** weightlessness, *Hericium erinaceus* polysaccharides, gastrointestinal dysfunction, gut microbiota, inflammation

## Abstract

**Background/Objectives**: Long-term spaceflight in a microgravity environment frequently results in gastrointestinal dysfunction, presenting substantial challenges to astronauts’ health. *Hericium erinaceus*, a plant recognized for its dual use as food and medicine, contains a key functional component called *Hericium erinaceus* polysaccharide (HEP), which is purported to promote gastrointestinal health. This study aims to investigate the protective effects of HEP against gastrointestinal disturbances induced by simulated weightlessness and to elucidate its regulatory mechanisms. **Methods**: Sprague Dawley rats subjected to a tail suspension model were administered either a standard diet or a diet supplemented with 0.125% HEP over a period of 4 weeks (the intake of HEP is approximately 157.5 mg/kg bw/d, n = 8), metagenomics and targeted metabolomics to investigate the effects of HEP on gastrointestinal hormone secretion disorders, gut microbiota dysbiosis, and intestinal barrier damage induced by simulated weightlessness. **Results**: Dietary supplementation with HEP was observed to significantly alleviate weightlessness-induced gastrointestinal hormone disruptions, enhancing motility and intestinal barrier function while reducing inflammation. In addition, HEP improved gut microbiota by boosting beneficial bacteria as *Oscillibacter sp.1-3*, *Firmicutes bacterium ASF500*, and *Lactobacillus reuteri*, while reducing harmful bacteria like *Escherichia coli* and *Mucispirillum schaedleri* at the species level. Furthermore, HEP altered the serum metabolic profile of the rats, reducing inflammation by upregulating the tryptophan metabolism pathway and enhancing the production of short-chain fatty acids. **Conclusions**: HEP effectively protects against gastrointestinal dysfunction induced by simulated weightlessness by regulating hormone secretion and maintaining intestinal homeostasis.

## 1. Introduction

Prolonged exposure to weightlessness during space flight leads to various physiological adaptive responses in the human body, including arrhythmia, osteoporosis, and alterations in gastrointestinal function [1,2,3]. Among these, complaints related to the digestive system have gradually become a concern, manifesting as varying degrees of indigestion, constipation, intestinal inflammation, and nutrient malabsorption in astronauts [4,5,6]. Current strategies to address these gastrointestinal symptoms associated with weightlessness primarily involve stimulating intestinal peristalsis through exercise [7] and administering medications such as antacids, gastric mucosal protectants, and motion sickness preventatives [8,9]. However, these interventions often yield limited results, and may change its turn-over in the human body when exposed to microgravity which ultimately discount its therapeutic effects. Consequently, finding ways to prevent or mitigate digestive system disorders in a weightlessness environment remains a critical challenge.

Changes in diet and environment can alter the microbiome of intestinal microorganisms, which in turn influence gastrointestinal function through their unique properties [10,11]. Firstly, normal gut microbiota forms a crucial physical barrier by tightly adhering to intestinal epithelial cells, preventing pathogen invasion. Beneficial bacteria, such as *Bifidobacteria*, also stimulate the secretion of mucin from intestinal epithelial cells, enhancing the chemical barrier of the gut [12,13]. Also, components of bacterial cell walls, such as lipopolysaccharides (LPS) from *Gram-negative bacteria*, can activate host immune responses [14]; however, an imbalance in gut microbiota can lead to these endotoxins entering the bloodstream and triggering inflammation [15]. Furthermore, the metabolic byproducts of gut microorganisms further affect host gastrointestinal function. For instance, short-chain fatty acids (SCFAs), produced by the metabolism of *Bifidobacteria* and *Bacteroides*, provide energy to intestinal cells and help maintain the integrity of the intestinal mucosa [16,17]; 5-hydroxytryptamine (5-HT) secreted by *Lactobacilli* stimulates intestinal smooth muscle contraction and promotes peristalsis [18,19], and as a neurotransmitter, 5-HT also influences appetite and satiety via the “gut–brain axis” [20]. Research has indicated that the structure of astronauts’ gut microbiota is disrupted in a weightless environment, with a noted decrease in beneficial bacteria such as *Lactobacillus* and *Bifidobacterium*, while conditional pathogens like *Clostridium* and *Pseudomonas* proliferate [21,22,23]. Additionally, the concentration of butyric acid, a key metabolite, is significantly reduced in the fecal matter of astronauts during space missions [22,24]. These findings underscore the critical role that intestinal microorganisms play in maintaining homeostasis, suggesting that targeted interventions to modulate gut flora may offer a promising approach to treat gastrointestinal dysfunction caused by weightlessness.

*Hericium erinaceus*, belonging to the *Basidiomycetes* phylum and *Hericiaceae* family, is a kind of homologous medicine and food [25,26]; its polysaccharide (HEP), extracted from the fruiting body, mycelium, and fermentation products, exhibits antioxidant, anti-tumor, and immunomodulatory properties [27,28]. As the primary active component of *Hericium erinaceus*, HEP has been shown to enhance gastrointestinal function by promoting the regeneration and repair of gastric mucosal epithelial cells, stimulating intestinal peristalsis, and restoring intestinal barrier homeostasis [29]. These spurred us to investigate whether HEP could mitigate the intestinal barrier dysfunction due to weightlessness, and if yes, the special mechanisms would be worthy of further exploration.

In this study, we aimed to simulate a weightlessness environment by establishing a tail suspension model in rats. We utilized research methods such as metagenomics and targeted metabolomics to investigate the effects of HEP on the improvement of gastrointestinal hormone secretion disorders, gut microbiota dysbiosis, and intestinal barrier damage induced by simulated weightlessness. Additionally, we sought to explore the relationship between gut microbiota and serum metabolites in rats during this process, in order to identify key bacterial genera and critical serum metabolites. Our findings provide new insights into the mechanisms through which HEP protects the intestine under weightless conditions and underscore the potential applications of HEP and gut microbiota modulation in counteracting the adverse effects of weightlessness.

## 2. Materials and Methods

### 2.1. Preparation of HEP Feed

The diets for the control group (CON) and the tail suspension model group (TSS) were prepared using a standard rat diet sourced from Spefox Biotechnology Co., Ltd. (Beijing, China). The diet for the HEP group was formulated by incorporating HEP powder (Xi’an Snott Biotechnology Co., Ltd., Xi’an, China) into the CON diet at a concentration of 0.125%. This dosage is based on the clinical recommendation for a daily intake of HEP for humans at 25 mg/kg bw/d. According to the “Conversion equivalent dosage ratio of surface area between humans and animals” (Supplementary Table S1), the calculated intake dosage for rats is 25 × 6.3 = 157.5 mg/kg bw/d. Considering the daily feed intake of rats is approximately 30–40 g/d, the final addition of HEP to the feed is set at 0.125%. Additionally, the content of nutrients such as starch and sucrose in the HEP group feed was reduced to ensure that both types of feed have equal energy content (Supplementary Table S2). Proximate analysis of the HEP powder was conducted by Pony Testing International Group (Beijing, China).

### 2.2. Animal Experiments

**Figure 1:**
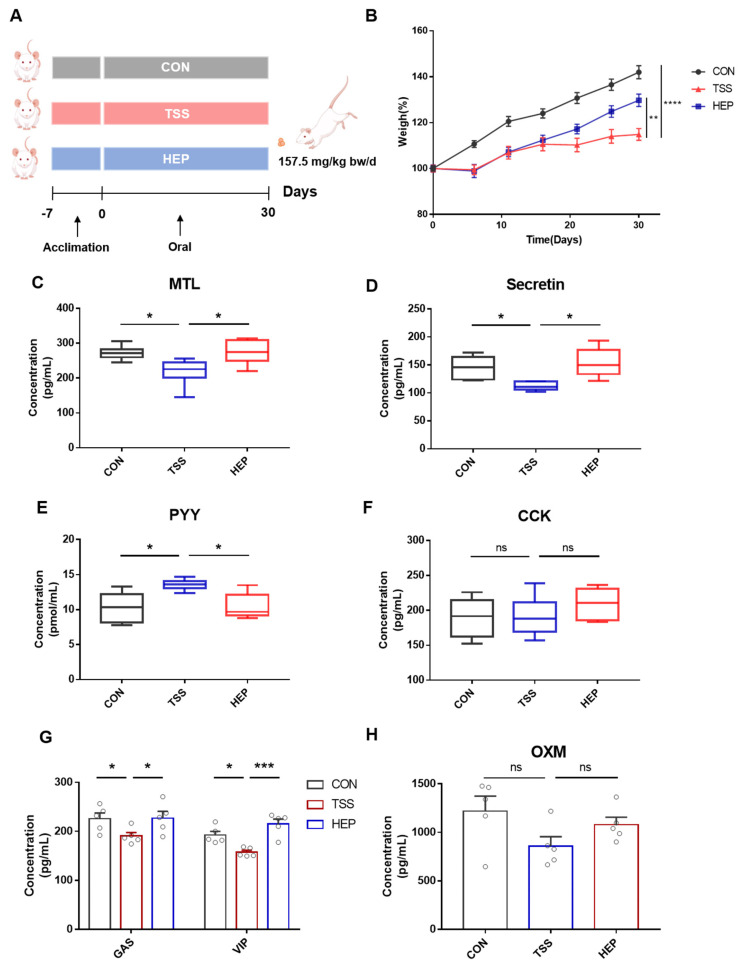
*Hericium erinaceus* polysaccharides (HEP) ameliorate weightlessness-induced gastrointestinal motility deficiency and digestive hormone secretion disorders in rats. (**A**) Experimental design of animal studies (n = 8); (**B**) percentage change in body weight of rats; (**C**–**F**) plasma levels of digestive hormones related to gastrointestinal motility (motilin, secretin, PYY, and CCK) in rats; (**G**,**H**) plasma levels of digestive hormones related to nutrient digestion physiology (gastrin, VIP, and OXM) in rats. Data are mean ± SEM. * *p* < 0.05, ** *p* < 0.01, *** *p* < 0.001 and **** *p* < 0.0001, ns indicates no significant change. For body weight change, a repeated measure two-way analysis of variance (ANOVA) was performed, while the other statistical analysis was performed with one-way ANOVA followed by Tukey’s multiple comparisons test.

A total of 24 male SD rats, aged 6 weeks and weighing 180–200 g were acquired from SPF Biotechnology Co., Ltd. (Beijing, China). The rats were randomly assigned to 3 groups: control (CON), tail suspension model (TSS), and HEP intervention (HEP), with 8 rats in each group (Figure 1A). All animals were acclimatized to laboratory conditions for 1 week, with ad libitum access to food and water in an SPF facility (temperature 22 ± 1 °C, humidity 60 ± 10%, 12 h light/dark cycle).

The formal experimental phase lasted 4 weeks. The TSS group was subjected to a tail suspension model based on the methodology described by Liang et al. [30]. The rats’ tails were disinfected with 75% alcohol before being suspended and secured to ensure that their hind limbs were elevated off the ground, maintaining a body angle of 30° relative to the horizontal, which simulates the microgravity environment experienced by astronauts in space by suspending the hind limbs of rats, putting them in a state similar to weightlessness. The rat forelimbs remained in contact with the bottom of the cage, allowing the rats to move freely and access food. Throughout the duration of the experiment, the rats maintained tail suspension until the end of the experiment.

Rats in the CON group received no intervention, while those in the HEP group were fed a special diet containing 0.125% HEP (based on the daily food intake of rats, the HEP intake level is approximately 157.5 mg/kg bw/d) while being suspended by their tails. The body weights of the rats were recorded weekly. After the experiment, blood samples were collected from the rats’ orbits, and intestinal tissues and fecal samples were obtained following euthanasia. All animal experiments were conducted in compliance with the Guidelines for Animal Experimentation of the China Astronaut Research and Training Center (Beijing, China) (Ethics reference number: ACC-IACUC-2021-014). All the protocols were approved by the Animal Ethics Committee of this institution and conducted according to ARRIVE standards (https://www.nc3rs.org.uk/arrive-guidelines, accessed on 27 January 2025).

### 2.3. Detection of Gastrointestinal Hormone Levels in Plasma

Whole blood samples were collected from the rats and treated with anticoagulant sodium heparin. The samples were then centrifuged at 3000 r/min for 10 min to obtain plasma. Following the instructions provided with the ELISA kits (Nanjing Jiancheng Bioengineering Institute Co., Ltd., Nanjing, China), we measured the levels of various gastrointestinal hormones and inflammatory markers in rat plasma, including motilin (MTL), secretin, peptide YY (PYY), gastrin (GAS), cholecystokinin (CCK), vasoactive intestinal polypeptide (VIP), glucagon-like peptide-1 (GLP-1), oxyntomodulin (OXM), calprotectin (CALP), and 5-hydroxytryptamine (5-HT).

In ELISA assays designed to detect gastrointestinal hormones and inflammatory markers in rat plasma, serum samples and standards are added to wells coated with antibodies, followed by incubation to promote hormone-antibody binding. After washing the wells, enzyme-conjugated secondary antibodies are introduced and incubated to form complexes. Subsequently, a substrate is added to initiate color development, and absorbance is measured after the reaction is terminated. The concentrations of the samples are then determined using a standard curve.

### 2.4. Histological Evaluation

Colon samples were fixed in 4% paraformaldehyde for 24 h and then embedded in paraffin for sectioning. Tissue slices of the colon were stained with hematoxylin and eosin (H&E). Digital images were captured at 100× or 400× magnification from 6 randomly selected microscopic fields for each tissue section.

### 2.5. Immunohistochemistry

Colonic sections were deparaffinized and rehydrated prior to blocking with 5% bovine serum albumin for 30 min at room temperature, followed by washing with PBS. The sections were then incubated overnight at 4 °C with primary antibodies GRP41 (Affinity, AF9075, Changzhou, China), GRP43 (Abcam, ab124272, Cambridge, UK), and Muc-2 (Santa Cruz, sc-515032, Dallas, TX, USA). After three washes in PBS, peroxidase-conjugated secondary antibodies (Abcam, ab150080, Cambridge, UK) were applied for 2 h at room temperature.

### 2.6. Inflammatory Cytokine Analysis

Cytokine levels in plasma, including diamine oxidase (DAO), D-lactic acid (D-Lac), LPS, and interleukins IL-2, IL-10, and IL-17, were detected by enzyme-linked immunosorbent assay (ELISA) kit (Nanjing Jiancheng Bioengineering Institute Co., Ltd., Nanjing, China) according to the instructions. Briefly, plasma samples and standards are added to antibody-coated wells, followed by incubation to facilitate cytokine–antibody binding. After washing, enzyme-conjugated secondary antibodies are added and incubated to form complexes. The substrate is then added for color development, and absorbance is measured after terminating the reaction. Cytokine concentrations are determined using a standard curve.

### 2.7. Metagenomics Analysis

Total DNA was extracted from each rat fecal sample using a DNA extraction kit (TianGen, Beijing, China). DNA samples that passed quality control were fragmented into approximately 350 bp segments using an ultrasonic nucleic acid fragmentor. The sequencing library was prepared using the TruSeq Nano DNA LT Library Prep Kit (Illumina, San Diego, CA, USA). The quality and quantity of the library were assessed using the Quant-iT PicoGreen dsDNA Assay Kit (Thermo, Waltham, MA, USA) on the Promega QuantiFluor fluorescence quantitative system.

Quality screening of raw double-end sequence data from high-throughput sequencing was conducted using Fastp (v0.20.0) to generate high-quality datasets (Clean data) suitable for downstream metagenomic analysis. High-quality sequences were corrected, and metagenome contigs were assembled. The MMseqs2 software (14-7e284) was employed to align region coverage at 90% for redundancy removal. Open reading frames (ORFs) within the contigs were identified using MetaGeneMark software (v.3.38), which also predicted the coding regions. Following de-redundancy, a non-redundant amino acid sequence set was generated. Finally, the amino acid sequences were functionally annotated using various commonly utilized databases to obtain abundance profiles of functional groups at different levels. All raw sequence data were archived in the NCBI Short Read Archive database under the Bioproject accession number PRJNA1214001.

### 2.8. Serum Metabolomics

Non-targeted metabolomics analysis was conducted using liquid chromatography-mass spectrometry (LC-MS, QTRAP^TM^ 6500+, AB Sciex Analytical Instrument Trading Co., Ltd., Framingham, MA, USA) to evaluate small molecule metabolites in rat serum samples. A 100 μL aliquot of serum was extracted with a methanol-acetonitrile solution (V:V = 2:1) containing an internal standard, vortexed for 30 s at 4 °C, and sonicated for 30 min. The samples were then incubated at −20 °C for 30 min to precipitate proteins. Following centrifugation at 13,000× *g* for 15 min at 4 °C, the supernatant was transferred to a sample vial for LC-MS/MS analysis.

The target compounds were chromatographically separated using a Vanquish ultra-high-performance liquid chromatograph (Thermo Fisher Scientific Co., Ltd., Waltham, MA, USA) on a Waters ACQUITY UPLC HSS T3 (2.1 mm × 100 mm, 1.8 μm) column. The mobile phase consisted of phase A (aqueous solution with 5 mmol/L ammonium acetate and 5 mmol/L acetic acid) and phase B (acetonitrile). The sample tray was maintained at 4 °C, and an injection volume of 2 μL was used.

The raw data were converted into mzXML format using ProteoWizard software (v5.0) and subsequently processed with the R package (utilizing XCMS v2.0) for peak identification, extraction, alignment, and integration. The data were matched against a secondary mass spectrometry database for compound annotation. Partial least squares discriminant analysis (PLS-DA) and orthogonal partial least squares discriminant analysis (OPLS-DA) were performed to identify differential metabolites, which were further analyzed using Student’s *t*-test, along with KEGG pathway enrichment analysis.

### 2.9. Determination of Short-Chain Fatty Acids

A 100 μL sample of rat serum was combined with 800 μL of distilled water, 200 μL of 50% concentrated sulfuric acid, and 1 mL of ether. The mixture was vortexed for 5 min to extract short-chain fatty acids (SCFAs). After centrifugation at 12,000× *g* for 10 min, anhydrous CaCl_2_ was added to the tube for dehydration. The supernatant was then carefully aspirated and filtered through a 0.22 μm nylon membrane. The analysis was conducted using a Shimadzu gas chromatograph equipped with a 30 m × 0.32 mm × 0.25 μm SH-Stabilwax-DA capillary column, with a solvent delay of 2 min. The initial temperature was set to 90 °C for 2 min, after which it was ramped at 15 °C/min to reach 220 °C, a temperature maintained for an additional 5 min. The detector temperature was set at 175 °C, and helium was used as the carrier gas at a flow rate of 1.0 mL/min. Data acquisition occurred in SIM mode using Shimadzu workstation software (LabSolutions LC/GC 5.93). Standard curves for acetic acid, propionic acid, and butyric acid were established, allowing for the quantification of SCFAs in the samples based on these standard curves.

### 2.10. Data Analysis

Statistical analyses were performed using Prism 9.0 software (GraphPad Software, San Diego, CA, USA). Data are presented as means ± SEM. Significant differences between two groups were assessed using a two-tailed unpaired Student’s *t*-test, while comparisons involving more than two groups were evaluated using one-way or two-way analysis of variance (ANOVA) followed by Tukey’s multiple comparisons test.

## 3. Results

### 3.1. HEP Improves the Disorder of Digestive Hormone Secretion in Rats Induced by Weightlessness

Compared to the control group, the weight of rats in the TSS group began to decrease on the 21st day after tail suspension and continued to worsen, while supplementation with HEP mitigated this weight loss (Figure 1B). Given that gastrointestinal hormone levels reflect gastrointestinal function and physiological changes in the digestive tract, we further analyzed the plasma concentrations of these hormones. The results indicated that the levels of motilin (MTL) and secretin, which are associated with gastrointestinal motility, were significantly lower in TSS group compared to CON group, and peptide YY (PYY) levels increased, while HEP supplementation effectively reversed these changes (Figure 1C–E). Additionally, the concentrations of gastrin (GAS) and vasoactive intestinal polypeptide (VIP), which are related to nutrient digestion, also decreased significantly in the TSS group, with HEP alleviating these downward trends (Figure 1G). No significant changes were observed in the plasma levels of cholecystokinin (CCK) and oxyntomodulin (OXM) (Figure 1F,H).

Upon morphological examination of the intestinal tissues in rats, no significant differences were found in the lengths of the jejunum and ileum among the three groups. In the CON and HEP groups, the boundaries of food retention were well defined, with intact colon and cecum and formed feces present in the colon. In contrast, the cecum of the TSS group was atrophied, with less distinct cecal nodules, food retention observed in all segments of the small intestine, and a lack of formed feces in the colon. In summary, HEP supplementation notably improved TSS-induced weight loss, decreased gastrointestinal motility, reduced defecation, and diminished nutrient digestion and absorption.

### 3.2. HEP Inhibits Intestinal Microflora Imbalance Caused by Weightlessness

**Figure 2:**
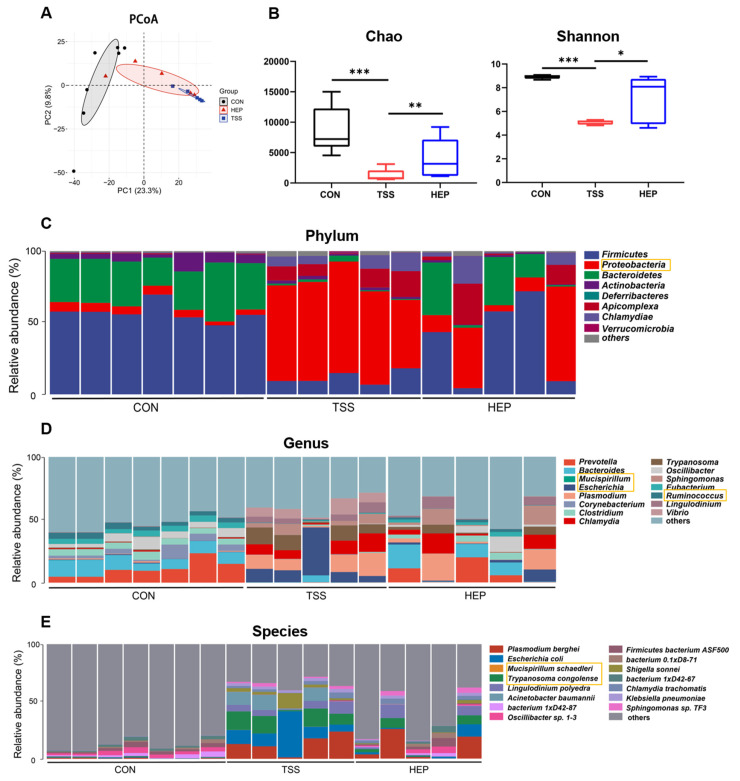
Effects of *Hericium erinaceus* polysaccharides (HEP) on gut microbiota in a weightlessness environment. (**A**) PCoA plot of the gut microbiota composition at the operational taxonomic unit (OTU) level from different rat groups (n = 5–7); (**B**) alpha diversity analysis of gut bacterial richness (Chao index) and diversity (Shannon index) from different rat groups (n = 5–7); (**C**–**E**) taxonomic distributions of gut bacterial composition at the phylum/genus/species levels (n = 5–7). Data are mean ± SEM. * *p* < 0.05, ** *p* < 0.01, and *** *p* < 0.001. The orange boxes indicate the species with significant changes. The statistical analysis was performed with one-way ANOVA followed by Tukey’s multiple comparisons test.

Previous studies have shown that weightlessness disrupts the intestinal microbiota [31]. To investigate whether HEP could mitigate TSS-induced gut microbiota dysbiosis, we performed metagenomic analyses of the colonic contents. PCA analysis based on operational taxonomic units (OTUs) revealed distinct clustering of the colonic microbiota composition in the TSS group compared to the CON group, indicating that tail suspension significantly altered the gut microbiota organization. However, the composition of the HEP group clustered similarly to the CON group, suggesting that HEP effectively suppresses the microbiota changes induced by weightlessness. Furthermore, compared to the CON group, the richness (Chao index) and community diversity (Shannon index) of colonic flora in the TSS group were significantly reduced, while both richness and diversity were notably improved in the HEP group (Figure 2A,B).

We further examined changes in the abundance of gut microbiota at the phylum, genus, and species levels. At the phylum level, HEP significantly attenuated the increase in abundance of *Proteobacteria* and *Chlamydiae* induced by TSS, while enhancing the decrease in *Firmicutes* and *Bacteroidetes* (Figure 2C). At the genus level, the relative abundances of *Bacteroides*, *Mucispirillum*, and *Sphingomonas* increased significantly in the TSS group, while HEP supplementation notably reduced the relative abundance of these genera (Figure 2D). At the species level, the TSS group exhibited significantly increased relative abundances of *Escherichia coli*, *Trypanosoma congolense*, and *Mucispirillum schaedleri*, while beneficial bacteria such as *Oscillibacter sp.1-3*, *Firmicutes bacterium ASF500*, and *Lactobacillus reuteri* decreased significantly. Following HEP intervention, these disorders were alleviated, with *Mucispirillum schaedleri* nearly absent, while the relative abundances of *Oscillibacter sp.1-3* and *Firmicutes bacterium ASF500*, which play important roles in maintaining human intestinal health, increased significantly (Figure 2E).

**Figure 3:**
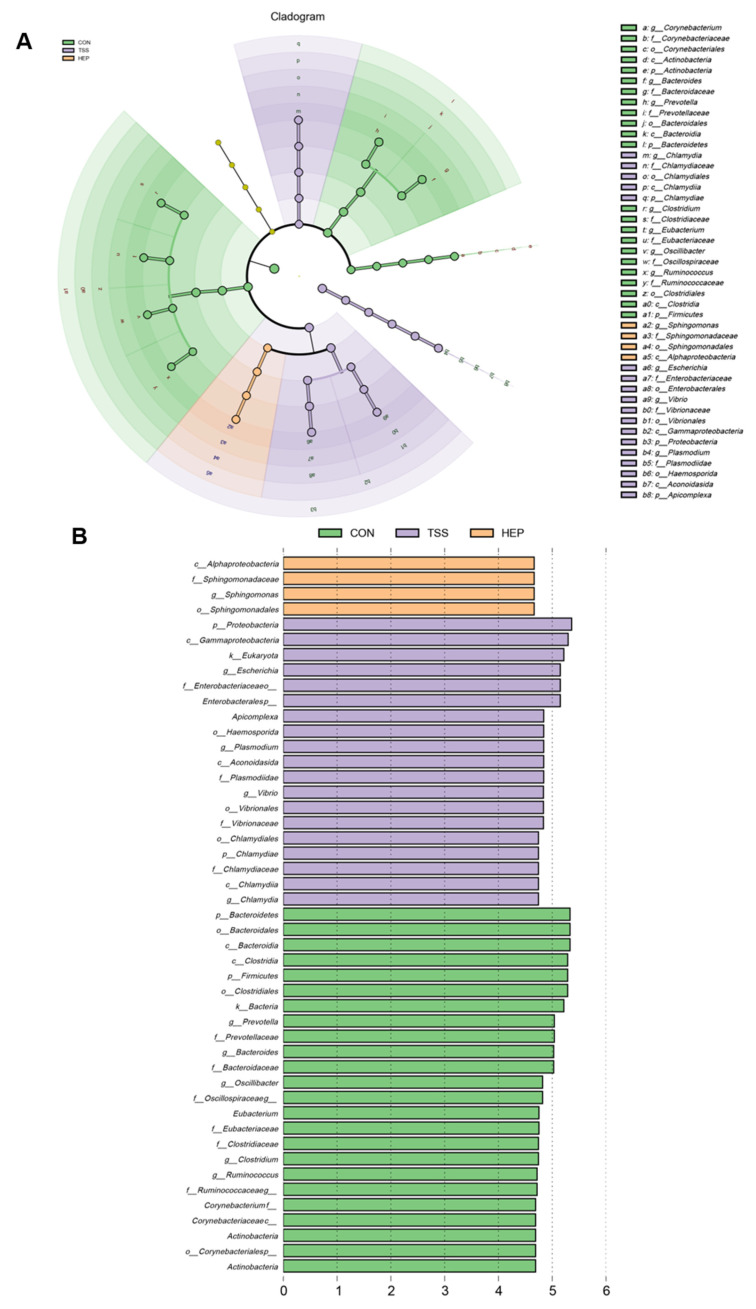
Effects of *Hericium erinaceus* polysaccharides (HEP) on rat intestinal characteristic bacteria and microbiota function. (**A**) LefSe phylogenetic branch diagram of characteristic bacteria in rat intestines (n = 5–7); (**B**) linear discriminant analysis (LDA > 2).

To clarify the effects of HEP on characteristic intestinal bacteria in rats, we conducted a LefSe analysis to assess microbial changes from the phylum to genus levels. The results have showed that the characteristic bacteria of CON group included *o_Bacteroidales*, *c_Clostridia*, *p_Firmicutes*, *g_Ruminococcus*, and *f_Prevotellaceae*. In contrast, the TSS group was characterized by *p_Proteobacteria*, *c_Gammaproteobacteria*, *k_Eukaryota*, *f_Enterobacteriaceae*, and *s_Escherichia_coli,* while the HEP group showed high levels of *c_Alphaproteobacteria*, *f_Sphingomonadaceae*, and *o_Sphingomonadales* (Figure 3A,B).

**Figure 4:**
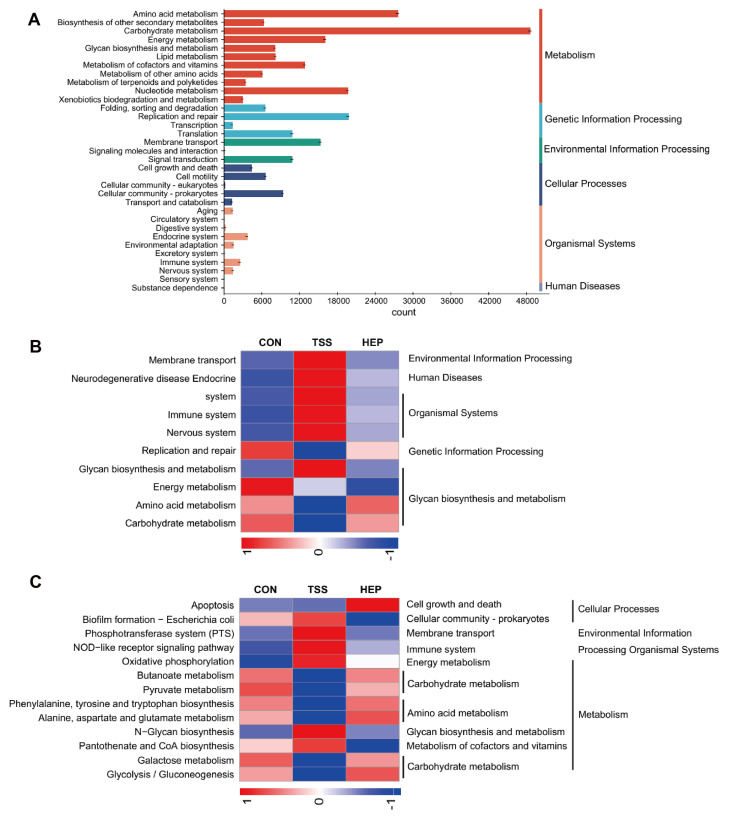
Effects of *Hericium erinaceus* polysaccharides (HEP) on the gut microbiota function of rats. (**A**) KEGG pathway annotation in 3 groups; (**B**) heatmap analysis of KEGG pathways at the second level of metabolism; (**C**) heatmap analysis of KEGG pathways at the third level of metabolism.

We then performed a KEGG metagenomic functional difference analysis to analyze the functional impact of HEP on gut microbiota. The samples were enriched in metabolic pathways related to amino acids, carbohydrates, and energy, as well as functions associated with the endocrine and immune systems. HEP supplementation significantly altered 25 pathways compared to the TSS group at the second enrichment level, with the 10 most significantly affected pathways including glycan biosynthesis, membrane transport, energy metabolism, amino acid metabolism, and the endocrine system (Figure 4A,B). The third enrichment level analysis revealed that HEP increased the enrichment of pathways related to pantothenate and coenzyme A biosynthesis, as well as oxidative phosphorylation in glycolysis. It also enhanced the enrichment of pathways for tyrosine and tryptophan biosynthesis and SCFA metabolism while reducing the enrichment of NOD-like receptor signaling pathways in the immune system, aligning them more closely with the CON group (Figure 4C).

### 3.3. HEP Alleviates Intestinal Barrier Disorder Induced by Weightlessness in Rats

**Figure 5:**
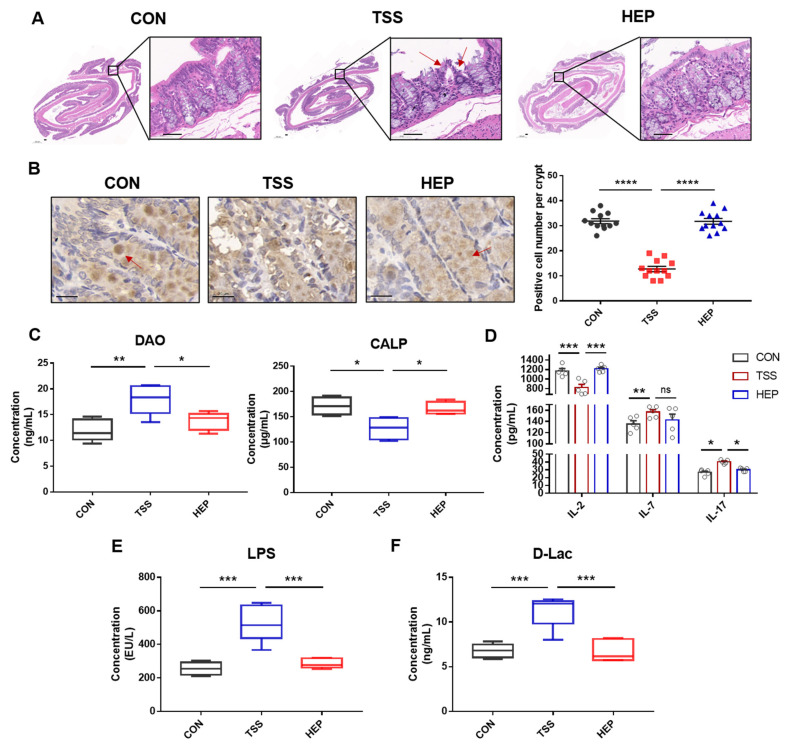
Effects of *Hericium erinaceus* polysaccharides (HEP) on weightlessness-induced intestinal barrier dysfunction in rats. (**A**) H&E staining of rat colon tissues (scale bar = 50 μm), the red arrow indicates the lesion; (**B**) immunohistochemical analysis of MUC2 (scale bar = 20 μm), red arrows indicate positive cells; (**C**) plasma levels of intestinal barrier function markers (DAO and CALP) in rats (n = 6) (**D**) plasma levels of interleukins (IL-2, IL-7, and IL-17) in rats (n = 6); (**E**,**F**) plasma levels of LPS and D-Lac (n = 6). Data are mean ± SEM. * *p* < 0.05, ** *p* < 0.01, *** *p* < 0.001, and **** *p* < 0.0001, ns indicates no significant change. Statistical analysis was performed with one-way ANOVA followed by Tukey’s multiple comparisons test.

We speculated that the preventive impacts of HEP on gastrointestinal disturbances caused by simulated weightlessness might be attributed to its potential to improve intestinal function. Investigation of intestinal morphology showed the colon mucosa of rats in the TSS group exhibited partial damage compared to CON group (Figure 5A), with mucus absent in the affected areas, a narrowed or absent mucosal layer, and degradation of the muscularis mucosa. Moreover, intestinal cavity pores were increased, and intestinal crypts were proliferated. These findings suggest that the tail suspension model simulating weightlessness led to abnormal development of the intestinal mucosa. In contrast, the mucosal layer in the HEP group remained intact, with compact cells, normalized intestinal crypts, restored muscularis mucosa thickness, and intact submucosal filamentous cells, indicating that HEP mitigated the morphological changes in colon tissue caused by weightlessness.

To further explore the impact of HEP on intestinal barrier function, we assessed the expression of MUC2 mucin, secreted by colon epithelial cells, through immunohistochemical analysis. HEP was found to improve the reduction of MUC2 caused by weightlessness (Figure 5B). Moreover, HEP supplementation alleviated the increase in plasma concentrations of diamine oxidase (DAO) and the decrease in calprotectin (CALP), which are markers of intestinal barrier function affected by weightlessness (Figure 5C). Inflammatory factor analysis revealed that the weightless environment led to reduced levels of interleukins IL-2, and increased levels of IL-7 and IL-17 in plasma (Figure 5D), with HEP supplementation reversing the imbalances of IL-2 and IL-17.

Increased permeability of the intestinal mucosal barrier allows endotoxins and D-lactic acid produced by intestinal bacteria to enter circulation through damaged areas of the mucosa. Measurement of lipopolysaccharide (LPS) and D-lactic acid (D-Lac) levels in plasma serves as an indicator of mucosal permeability. As shown in Figure 5E,F, plasma levels of D-Lac and LPS were significantly elevated in the TSS group, while HEP supplementation effectively reduced these levels, indicating that HEP can alleviate damage to intestinal barrier function, increased permeability, and the resultant intestinal inflammation caused by the simulated weightlessness environment.

### 3.4. HEP Improves the Serum Metabolic Profile Disorder Induced by Weightlessness in Rats

**Figure 6:**
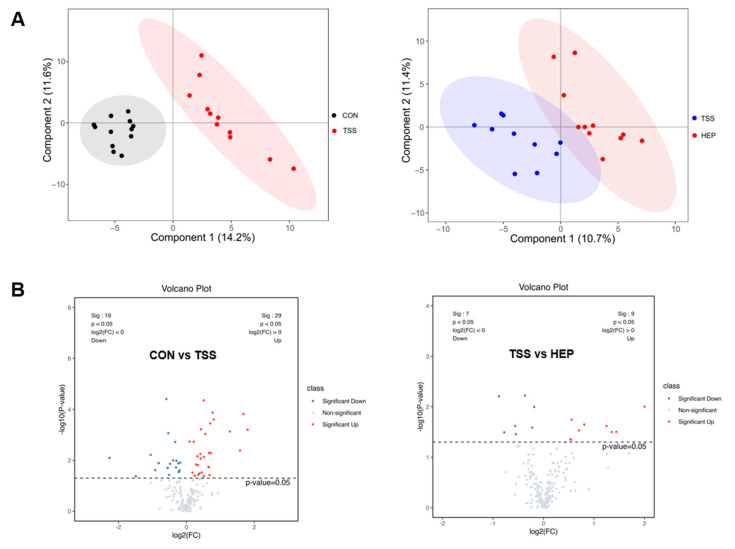

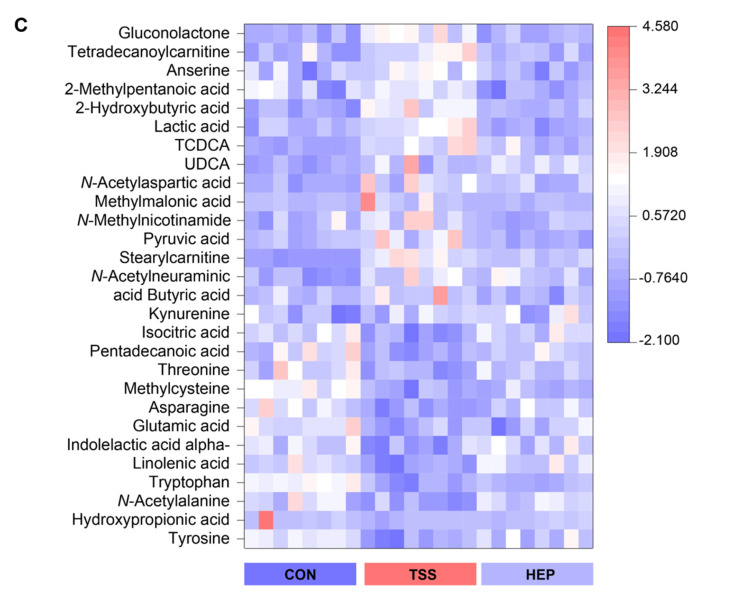
Effects of *Hericium erinaceus* polysaccharides (HEP) on rat serum metabolomics in a weightlessness environment. (**A**) Principal component analysis (PCA) of off-target metabolic profiling of rat serum; (**B**,**C**) volcano plots and heatmaps of significantly altered metabolites in rat serum.

To further elucidate the regulatory effects of HEP on inflammatory pathways in the host, we conducted a non-targeted metabolomics analysis of serum metabolites in rats following HEP intervention. Principal component analysis (PCA) was employed to assess the overall differences in serum metabolite compositions between the CON group and the TSS group, and between the TSS group and the HEP group. As illustrated in Figure 6A, the metabolite profiles of the CON and HEP groups were distinctly separated from that of the TSS group, indicating that the weightless environment significantly disrupted the serum metabolic profile of the rats, a disruption that HEP supplementation could effectively mitigate. Further statistical analysis revealed that compared to the CON group, the TSS group exhibited an upregulation of 29 serum metabolites and a downregulation of 19 metabolites, resulting in notable clustering differences. Particularly, there was a significant increase in metabolites such as pyruvate, *N*-acetylceramide, and lactate, alongside a decrease in tryptophan, cysteine, glutamate, and tyrosine. Conversely, HEP intervention in the tail suspension model resulted in an upregulation of nine metabolites and a downregulation of seven metabolites. Notably, HEP significantly inhibited the increases in pyruvate and lactate, as well as the decreases in tryptophan and tyrosine induced by weightlessness (Figure 6B,C).

**Figure 7:**
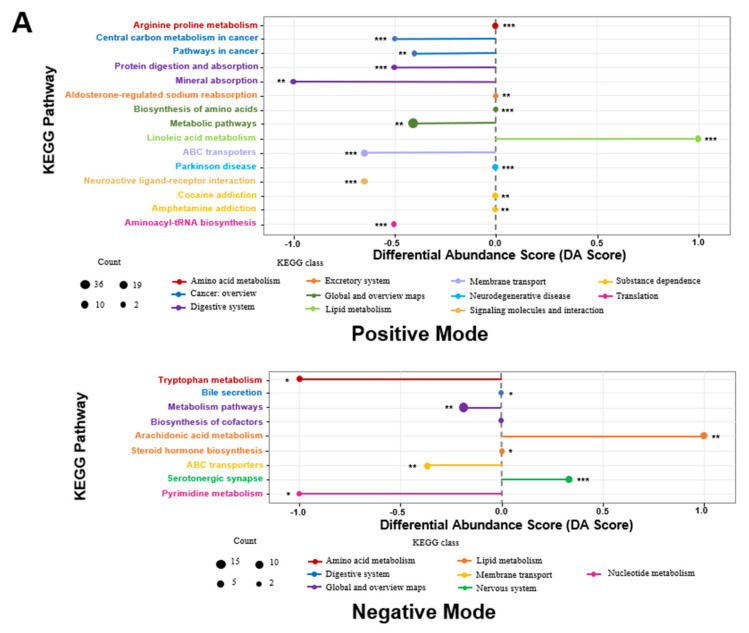

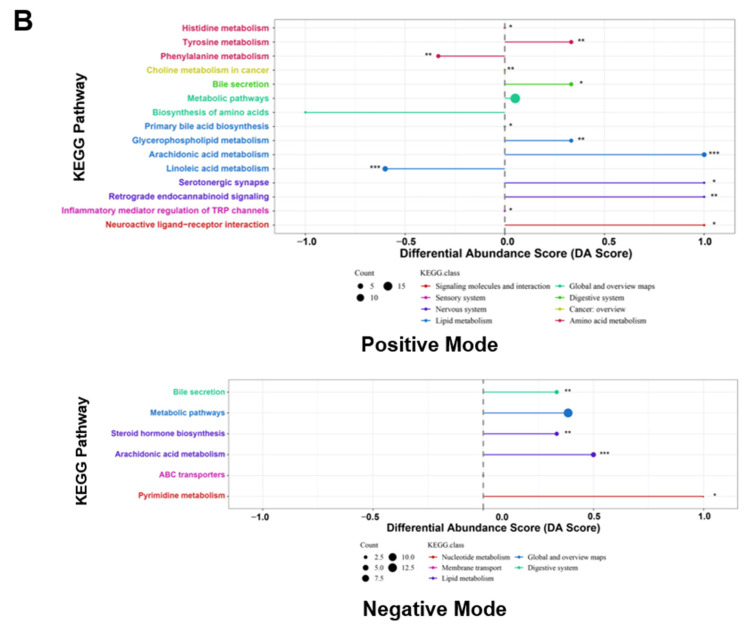
Effects of *Hericium erinaceus* polysaccharides (HEP) on the functional differences of serum metabolites in rats under weightlessness. (**A**) DA score and pathway enrichment analysis of KEGG metabolic pathways between the CON group and the TSS group; (**B**) DA score and pathway enrichment analysis of KEGG metabolic pathways between the TSS group and the HEP group. The length of the line segments represents the absolute value of the DA score, and the size of the dots corresponds to the number of differential metabolites, with larger dots indicating a greater number of metabolites. * *p* < 0.05, ** *p* < 0.01 and *** *p* < 0.001.

The differential abundance score (DA score) and pathway enrichment analysis of KEGG metabolic pathways revealed that the weightless environment adversely affected pathways associated with amino acid metabolism, digestive and excretion systems, membrane transport, cancer, and neurodegenerative diseases. A positive DA score indicates an upregulation of differential metabolite expression within the pathway, while a negative score indicates downregulation. Notably, the weightlessness treatment significantly upregulated the linoleic acid metabolism and serotonin synaptic metabolic pathways, while downregulating pathways related to neuroactive ligand–receptor interactions, protein digestion and absorption, tryptophan metabolism, pyrimidine metabolism, and ABC transport (Figure 7A). These alterations reflect a diminished capacity for nutrient digestion and absorption, reduced substance synthesis ability, and abnormal fat and protein metabolism, which may contribute to physiological issues such as bone loss and muscle atrophy associated with space weightlessness. In comparison to the TSS group, the HEP group significantly downregulated the serotonin synaptic metabolic pathway while upregulating pathways related to tryptophan metabolism, pyrimidine metabolism, retrograde endocannabinoid signaling, ABC transport, and primary bile acid synthesis (Figure 7B). The changes in the tryptophan signaling pathway and serotonin synaptic metabolism align with previous findings indicating that alterations in gut microbiota can impact tryptophan and inflammatory pathways. This suggests that HEP supplementation can substantially reverse the effects of weightlessness on abnormal serum metabolism and improve the intestinal inflammatory response in rats.

## 4. Discussion

**Figure 8:**
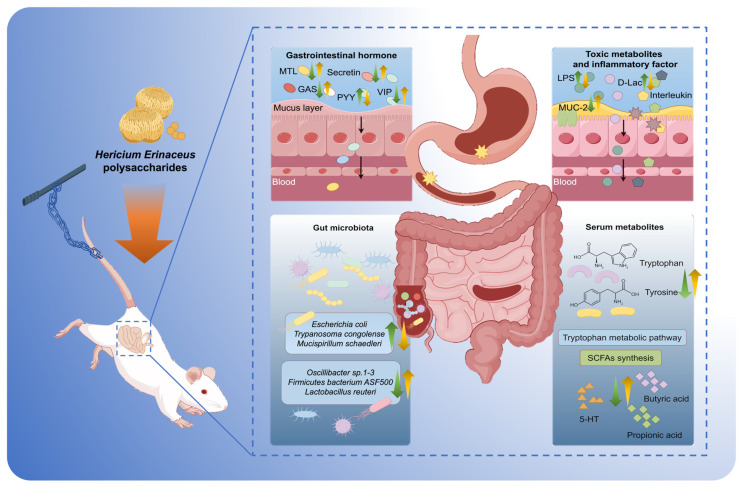
Schematic illustration of *Hericium erinaceus* polysaccharides (HEP) improving intestinal microbiome and intestinal barrier dysfunction induced by simulated weightlessness in rats (the green arrows represent the change trends of hormone, microbiota, and metabolites in rats with tail suspension treatment; the yellow arrows represent the change trends after HEP intervention).

In recent years, the surge in global investments in space exploration has resulted in a significant rise in long-duration manned space missions (lasting 365 days or more) as well as the emergence of civilian space flights [32,33]. Consequently, the imperative to protect astronaut health during extended spaceflights has become increasingly critical. Weightlessness is known to cause digestive problems such as indigestion and constipation, necessitating a comprehensive understanding of its pathogenesis and the development of effective dietary interventions. In this study, we demonstrated that dietary supplementation with the food-drug homolog HEP can mitigate the digestive dysfunction induced by simulated weightlessness in a rat tail suspension model, which is mainly reflected in the restoration of gastrointestinal hormone secretion, improvement of intestinal barrier function, and modulation of inflammatory responses. Notably, our findings indicate that HEP can regulate the composition of intestinal microbiota and functional genomes, alter the serum metabolic profile, and particularly influence the tryptophan metabolic pathway and the production of SCFAs. Our findings suggest for the first time that HEP affects the secretion of gastrointestinal hormones by modulating gut microbiota and metabolic processes, thereby alleviating weightlessness-induced gastrointestinal motility deficiencies and improving inflammation-related damage to intestinal barrier function (Figure 8). This not only provides a novel strategy for maintaining health in space but also highlights the potential for exploring the unique physiological stress responses in space through the integration of food and medicine.

In the weightless environment, the absence of a hydrostatic pressure gradient leads to abnormal fluid distribution in the body, significantly affecting neural and endocrine regulatory mechanisms [34,35]. For instance, mechanical receptors in the gastrointestinal tract may transmit abnormal signals to the nervous system due to altered food distribution and changes in gastrointestinal motility under weightless conditions [30]. These signal alterations can disrupt neuro-endocrine systems, such as the hypothalamus–pituitary–target gland axis, resulting in the dysregulation of digestive hormones like GAS, MTL, secretin, and OXM [36]. This disruption has been substantiated by various studies, encompassing both animal experiments and observations of astronauts during space missions [5,37,38]. PYY, predominantly secreted by L cells in the distal intestine, typically functions to inhibit appetite and reduce gastrointestinal motility and gastric acid secretion [39,40]. However, in a microgravity environment, the body’s stress response and regulatory changes in the nervous system can stimulate intestinal endocrine cells, leading to increased PYY secretion. Additionally, alterations in gut microbiota and gastrointestinal dysfunction may further enhance PYY release [41]. VIP, involved in vasodilation and gastrointestinal functions, may also increase under weightless conditions to counteract abnormal blood perfusion that affects cardiovascular and gastrointestinal regulation [42]. In this study, HEP, recognized for its diverse functions, significantly improved the secretion of gastrointestinal hormones. Its regulatory mechanisms may involve two key aspects: Firstly, HEP can support the growth of beneficial bacteria, such as *Bifidobacteria* and *Lactobacilli* [43], thereby optimizing the intestinal microbiota composition and regulating gastrointestinal hormone secretion through the “microbiota–gut–brain” axis. Secondly, HEP exhibits antioxidant properties that mitigate oxidative stress-induced damage to hormone-secreting cells in the gastrointestinal tract [43], thereby restoring normal hormone secretion patterns.

The role of intestinal microbiota in maintaining the homeostasis of the intestinal barrier is well documented, and exposure to weightlessness can significantly disrupt this microbial balance. NASA’s twin astronaut study revealed notable alterations in the intestinal microbiota of astronauts in space, characterized by an increase in *Firmicutes* and a decrease in *Bacteroidetes* [44]. Similar dysbiosis has been observed in astronauts aboard the International Space Station, characterized by reduced populations of protective bacteria like *Bifidobacteria* and *Lactobacilli* and the emergence of pathogenic bacteria such as *Escherichia coli* and *Clostridium*. These alterations typically resolve upon the return to a normal gravity environment [45,46]. Our study indicated that HEP effectively reduced the abundance of *Escherichia coli*, *Trypanosoma congolense*, and *Mucispirillum schaedleri* induced by weightlessness, while concurrently increasing the relative abundance of *Oscillibacter sp.1-3* and *Firmicutes bacterium ASF500* [47]. Notably, *Mucispirillum schaedleri* exhibited the most significant changes, suggesting its potential role as a biomarker for weightlessness-induced dysbiosis. This genus, which is known for its presence in the intestinal mucus layer, has been associated with inflammatory responses in conditions like Crohn’s disease and type 2 diabetes (T2DM). Previous research indicates that bioactive substances can reduce *Mucispirillum schaedleri* abundance and improve intestinal inflammation. For example, black bean polysaccharides alleviate enteritis in T2DM rats by decreasing this bacterium and enhancing linoleic acid metabolism [48,49]. Similarly, quercetin mitigates celiac disease-related inflammation by reducing *Mucispirillum schaedleri* and balancing immune cells [34]. In this study, HEP significantly reduced the relative abundance of *Mucispirillum schaedleri* and alleviated the inflammatory response in the intestine of rats, which may be related to HEP’s antioxidant activity. On the one hand, HEP inhibited the growth and expansion of *Mucispirillum schaedleri* by enhancing the host’s immune defense mechanism; on the other hand, HEP reduced the pathogenic potential of *Mucispirillum schaedleri* by regulating the intestinal microenvironment, such as increasing the production of SCFAs and improving intestinal barrier function [50,51].

Apart from gut microbiota, intestinal motility and barrier function are critical factors sustaining intestinal homeostasis, and dysfunction in these areas is a key symptom of weightlessness [52]. Our study found that HEP significantly improved intestinal barrier damage caused by simulated weightlessness. This was shown by increased intestinal crypt height, muscle layer width, MUC2 mucin expression, and levels of DAO and CALP, along with normalized inflammation indicators. In a weightless environment, the human body experiences oxidative stress, along with mechanical and chemical stimulation of intestinal epithelial cells, which results in increased intestinal permeability and the induction of inflammatory responses. These conditions facilitate the release of proinflammatory cytokines and the activation of proteases, leading to the degradation of MUC2 mucin and the intestinal mucosa [53]. Consequently, LPS and D-Lac can enter the bloodstream and raise their plasma levels [54,55]. HEP can enhance the antioxidant capacity of intestinal epithelial cells, reduce the damage to cells caused by oxidative stress, reduce intestinal permeability, promote the synthesis and secretion of anti-inflammatory factors IL-2 and IL-10, reduce inflammatory reactions, and alleviate the expression of MUC2 mucin and the disordered regulation of the levels of intestinal barrier function markers DAO, CALP, LPS, and D-Lac. In addition, HEP regulates the gut microbiota and increases the relative abundance of beneficial flora. These beneficial bacteria produce SCFAs, which support mucin synthesis and enhance mucosal integrity, protecting the intestinal barrier.

**Figure 9:**
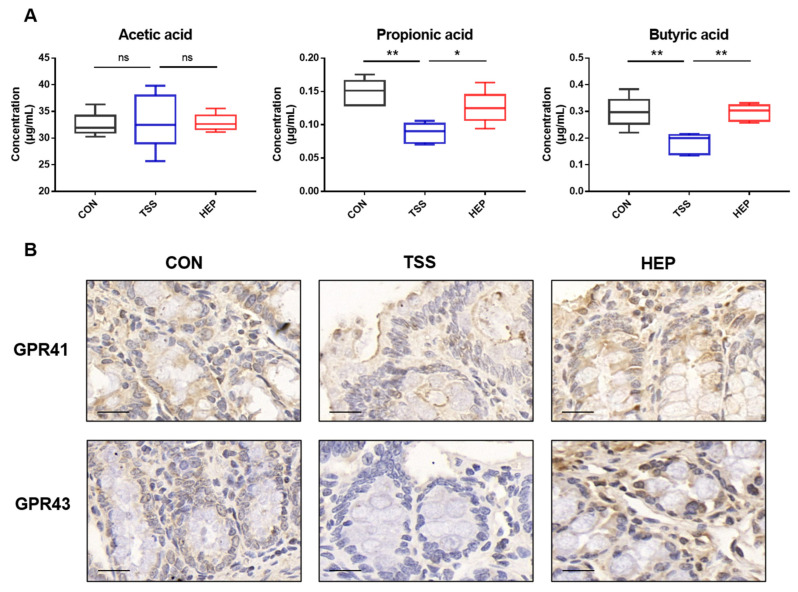

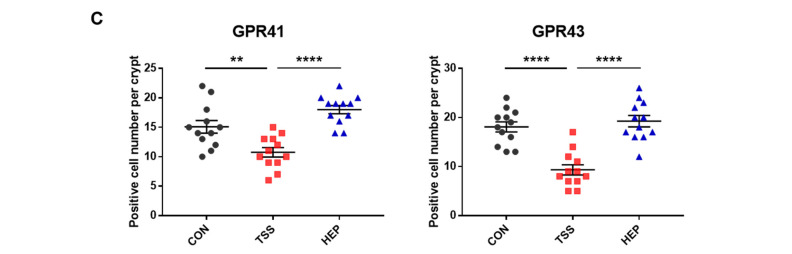
*Hericium erinaceus* polysaccharides (HEP) on the content of short-chain fatty acids and their receptor expression in rats. (**A**) Serum levels of SCFAs (acetic acid, propionic acid, and butyric acid) in rats. (**B**) Immunohistochemical staining of GPR41 and GPR43 in the colon of rats (scale bar = 20 μm). (**C**) Percentage of cells positive for GPR41 and GPR43. Data are mean ± SEM. * *p* < 0.05, ** *p* < 0.01, and **** *p* < 0.0001. ns indicates no significant change. Statistical analysis was performed with one-way ANOVA followed by Tukey’s multiple comparisons test.

We used metagenomics and serum non-targeted metabolomics to investigate how HEP influences host metabolism by modulating gut microbiota. KEGG analysis revealed that HEP supplementation significantly impacted various metabolic pathways, including glycan biosynthesis, energy metabolism, amino acid metabolism, and endocrine system functions. Notably, HEP boosted tryptophan biosynthesis and SCFA metabolism, increasing *Ruminococcaceae* abundance, which produces SCFAs. In this study, it was found that HEP supplementation can restore the levels of SCFAs (propionic acid and butyric acid) in the serum of rats in the TSS group, and increase the expression of SCFA receptors GPR41 and GPR43 in the rat colon (Figure 9). SCFAs provide energy, regulate intestinal pH, and enhance peristalsis, improving metabolic health [17]. HEP also reduced the enrichment of NOD-like receptor signaling pathways in the immune system and pathways related to *E. coli* biofilm formation, alleviating intestinal inflammatory responses and stabilizing the intestinal microbiota. Serum metabolomics analysis showed that HEP significantly inhibited increases in pyruvate and lactate and decreases in tryptophan and tyrosine caused by weightlessness. Pyruvate and lactate are products of glycolysis, and their increased levels may reflect disrupted energy metabolism [56]. Tryptophan and tyrosine are important amino acids involved in various physiological processes, including neurotransmitter synthesis [57,58]. The regulation of these serum metabolites by HEP may relate to its impact on intestinal microbiota, which can modulate serum tryptophan levels by affecting the tryptophan metabolic pathway, thereby influencing neurotransmitter synthesis and the physiological function of the host.

Overall, our findings indicate that HEP modulates the composition and function of intestinal microbiota, thereby influencing the host’s energy, amino acid, and SCFA metabolism. This modulation results in enhanced serum metabolite profiles and an improved overall metabolic status in rats subjected to weightlessness. Nonetheless, this study is subject to certain limitations, particularly the necessity for a more comprehensive investigation into the specific molecular mechanisms by which HEP regulates intestinal microbiota. Future research should prioritize conducting detailed experiments to further elucidate the effects of HEP on intestinal microbiota and host metabolism.

## 5. Conclusions

In this study, we demonstrate that dietary supplementation with HEP effectively protects against gastrointestinal dysfunction induced by simulated weightlessness. HEP alleviates disruptions in gastrointestinal hormone secretion, enhances intestinal barrier function, and mitigates microbiota dysbiosis in rats. Furthermore, HEP modifies the serum metabolic profile by alleviating inflammation, upregulating the tryptophan metabolism pathway, and increasing the production of short-chain fatty acids. These findings suggest a novel approach for the prevention and treatment of gastrointestinal disturbances experienced by astronauts in space environments.

## Data Availability

Data are contained within the article.

## Supplementary Materials

The following supporting information can be downloaded at: https://www.mdpi.com/article/10.3390/nu17040724/s1, Table S1: Conversion equivalent dosage ratio of surface area between humans and animals; Table S2: The macronutrient composition of CON and HEP group diet.

## Abbreviations

The following abbreviations are used in this manuscript:

CALP	Calprotectin
CCK	Cholecystokinin
DAO	Diamine oxidase
D-Lac	D-Lactic acid
GAS	Gastrin
GLP-1	Glucagon-like peptide-1
H&E	Hematoxylin and eosin
HEP	Hericium erinaceus polysaccharide
LPS	Lipopolysaccharides
MTL	Motilin
OXM	Oxyntomodulin
PCA	Principal component analysis
PYY	Peptide YY
SCFAs	Short-chain fatty acids
VIP	Vasoactive intestinal polypeptide
5-HT	5-hydroxytryptamine
