# Supplementary material for: Alteration of Gastrointestinal Function and the Ameliorative Effects of Hericium erinaceus Polysaccharides in Tail Suspension Rats
Source: Nutrients. 2025 Feb 18;17(4):724. doi: 10.3390/nu17040724 (PMC11858084; doi:10.3390/nu17040724)
Supplement: Supplementary file 1 [file nutrients-17-00724-s001.zip › nutrients-3473370-supplementary.pdf]

Supplementary Materials

Alteration of Gastrointestinal Function and the Ameliorative Effects of *Hericium erinaceus* Polysaccharides in Tail Suspension Rats

Peng Zang [1,2], Pu Chen [2], Junli Chen [2], Jingchao Sun [2], Haiyun Lan [2], Haisheng Dong [2], Wei Liu [2], Nan Xu [2], Weiran Wang [2], Lingwei Hou [2], Bowen Sun [2], Lujia Zhang [2], Jiaqiang Huang [3,*], Pengjie Wang [3], Fazheng Ren [1,3] and Siyuan Liu [3,*]

This file includes:

Table S1. Conversion equivalent dosage ratio of surface area between humans and animals;

Table S2. The macronutrient composition of CON and HEP group diet.

Correspondence: Department of Nutrition and Health, China Agricultural University, Beijing 100193, China.
E-mail: jqhuang@cau.edu.cn; Tel.: +86-18810281834;
siyuan.liu@cau.edu.cn; Tel.: +86-18825133260

Table S1. Conversion equivalent dosage ratio of surface area between humans and animals

	**Mouse (20 g)**	**Rat (200 g)**	**Cavy (400 g)**	**Rabbit (1.5 kg)**	**Cat (2.0 kg)**	**Dog (12 kg)**	**Human (70 kg)**
Mouse (20 g)	1.00	7.00	12.25	27.80	29.70	124.20	387.90
Rat (200 g)	0.14	1.00	1.74	3.90	4.20	17.80	56.00
Cavy (400 g)	0.08	0.57	1.00	2.23	2.40	4.20	31.50
Rabbit (1.5 kg)	0.04	0.25	0.44	1.00	1.08	4.50	14.20
Cat (2.0 kg)	0.03	0.23	0.41	0.92	1.00	4.10	13.00
Dog (12 kg)	0.008	0.06	0.10	0.22	0.23	1.00	8.10
Human (70 kg)	0.0026	0.018	0.031	0.07	0.078	0.82	1.00

Table S2. The macronutrient composition of CON and HEP group diet.

Composition (on a 90% dry matter basis)	Unit	CON diet	HEP diet
HEP	g/kg	0	1.25
Crude protein	g/kg	184.2	177
Crude fat	g/kg	45	38.5
Crude fiber	g/kg	42	39
Ash	g/kg	69	67
Carbohydrates	g/kg	732	687
Vitamin B12	mg/kg	0.037	0.036
Biotin	mg/kg	0.177	0.185
Nicotinic Acid	mg/kg	31.1	31.1
Sodium	g/kg	2.51	2.55
Calcium	g/kg	10.2	10.2
Potassium	g/kg	7.34	7.31
Glutamic Acid	%	2.7	2.68
Aspartic Acid	%	1.19	1.24
Proline	%	0.93	0.93
Alanine	%	1.16	1.2
